# Determinants of women’s autonomy in healthcare decision-making in Somalia: evidence from the 2020 demographic and health survey

**DOI:** 10.1186/s12939-026-02804-3

**Published:** 2026-02-26

**Authors:** Hamze G. Dahir, Ilham Muse Nour, Hakima Abdirahman Ahmed, Abdirashid M. Yousuf, Abdisalam Hassan Muse, Samakaab Baashe Ahmed

**Affiliations:** 1 School of Public Health and Nutrition, College of Health Sciences, Amoud University, Borama, Awdal, 25263 Somalia; 2 School of Medicine, College of Health Sciences, Amoud University, Borama, Awdal, 25263 Somalia; 3 School of Medical Laboratory, College of Health Sciences, Amoud University, Borama, Awdal, 25263 Somalia; 4 Research and Innovation Center, Amoud University, Borama, 25263 Somalia; 5 Faculty of ICT, Amoud University, Borama, 25263 Somalia

**Keywords:** Women’s autonomy, Healthcare decision-making, Somalia, Relational autonomy, Multilevel analysis, Gender equity, Negotiated agency

## Abstract

**Background:**

Women’s healthcare decision-making autonomy in Somalia is essential for empowerment and viewed as a fundamental human right. In fragile states, local socio-cultural structures and gatekeeping norms uniquely influence the typical drivers of agency, such as education and wealth. This study provides the first national analysis of the levels and determinants of healthcare autonomy among married women using data from the 2020 Somalia Demographic and Health Survey (SDHS).

**Methods:**

We analyzed a nationally representative sample of 7,492 married women. A multilevel mixed-effects binary logistic regression model was employed to account for the hierarchical structure of the data, with individuals nested within primary sampling units (PSUs). Autonomy was conceptualized using a relational framework and defined as meaningful participation in healthcare decisions (either alone or jointly).

**Results:**

Overall, 67.1% of women reported healthcare autonomy. A “Permission Paradox” was identified: women reporting that obtaining permission was a major barrier had significantly higher odds of autonomy (AOR = 1.79; 95% CI: 1.43–2.23; *p* < 0.001), suggesting that agency in this context is a negotiated process. Conversely, an “Education Anomaly” was observed, whereby secondary education was associated with a 41% reduction in the odds of autonomy (AOR = 0.59; *p* = 0.001). Regional context emerged as the strongest predictor; women in Bakool had nearly 4.5 times higher odds of autonomy (AOR = 4.47; *p* = 0.001) compared to those in Awdal.

**Conclusion:**

Healthcare autonomy in Somalia represents a negotiated agency embedded in geospatial heterogeneity. One-third of women remain entirely excluded from health-related decisions. Policy responses should move beyond individualistic models toward decentralized, culturally grounded interventions that address structural gatekeeping and regional disparities to advance meaningful health equity.

**Trial registration:**

Not applicable.

## Background

Fundamentally, women’s autonomy in healthcare decision-making is a critical element of their overall empowerment and a fundamental human right [1, 2] This autonomy represents a woman’s capacity to independently make informed choices about her own body and health, free from coercion or undue influence from her partner, family, or community [3]. Indeed, this concept underscores the significance of bodily integrity, self-determination, and gender equality, asserting that women’s autonomy in healthcare is crucial for their well-being and that of their families and communities [4, 5]. It is a complex construct shaped by various individual, household, and societal factors, including a woman’s age, education, employment status, wealth, and the educational and occupational characteristics of her partner [6, 7]. Furthermore, Broader contextual factors like residential location, region, and cultural norms significantly influence women’s healthcare decision-making autonomy [8]. Understanding these trends is crucial for creating effective policies and interventions to enhance women’s health and well-being.

The ability of women to make autonomous decisions regarding their health is a cornerstone of public health and a significant indicator of gender equality [8, 9]. Consequently, when women are empowered to make their own choices, it leads to improved maternal and child health outcomes, increased use of essential health services, and greater overall well-being for families and communities [5, 6]. However, in many parts of the world, women’s autonomy is constrained by a variety of socio-cultural and economic factors. The landscape of women’s healthcare decision-making autonomy varies significantly across different global contexts, influenced by varying access to education and economic opportunities [8, 10]. In Somalia, women’s access to education is influenced by specific socio-cultural and geographical factors, requiring a context-specific understanding of agency that acknowledges local social structures.

Globally, the prevalence of women’s autonomy in healthcare decision-making varies significantly. For instance, a study encompassing 57 countries revealed that in some nations in sub-Saharan Africa, less than 40% of women participate in decisions about their own healthcare, whereas in parts of Europe and Latin America, this figure exceeds 80% [10]. This disparity highlights the profound impact of socio-cultural and economic contexts. Within the African continent, the situation is particularly concerning. While progress has been made, women’s autonomy remains low across many regions. For example, a meta-analysis found the lowest prevalence in Nigeria at 36.16%, while Ethiopia reported a higher but still suboptimal rate of 61.36% [11]. In East Africa, a study among youth (15–24 years) in 11 countries found that 68.37% had autonomy in healthcare decision-making [11]. Factors consistently associated with higher autonomy across these regions include older age, higher education levels for both the woman and her partner, employment, and higher household wealth [7, 11]. For example, a study in Senegal showed that women with a higher level of education were 5.5 times more likely to have decision-making autonomy [12], and in Tanzania, employed women were more likely to be autonomous in their healthcare decisions [4]. The 2020 Somalia Health and Demographic Survey (SHDS) reveals that women in Somalia face significant barriers to accessing health services, including the need for permission from family members and a low level of autonomy [13]. Additionally, 46.5% of Somali women lack education, further exacerbating their limited autonomy. Cultural norms favoring men as primary decision-makers further exacerbate this situation [13].

The protracted conflict and humanitarian crisis in Somalia have had a devastating impact on the country’s health system, leaving women and children particularly vulnerable [14]. In this context, the 2019 Somali Health and Demographic Survey (SHDS) provides national data on the health and demographic status of the Somali population. Somalia has a population of approximately 17 million people, with a high total fertility rate of around 6.2 births per woman and a relatively low contraceptive prevalence rate of 16% among married women. The maternal mortality ratio is estimated at 692 deaths per 100,000 live births, highlighting significant challenges in maternal healthcare access and quality [13]. Somalia’s family planning efforts, driven by the National Reproductive Health Strategy, face significant challenges from insecurity, limited infrastructure, and cultural beliefs despite utilizing community health workers and NGOs. Understanding women’s healthcare autonomy through data like the 2020 SDHS is crucial for effective policy and improved health outcomes in this unstable region [13].

This study provides the first comprehensive analysis of trends and determinants of women’s healthcare decision-making autonomy in Somalia using the 2020 SDHS. By focusing on a context largely absent from the women’s empowerment literature, it generates novel, country-specific empirical evidence on the structural and socio-demographic drivers of autonomy. The findings offer a robust evidence base for designing targeted, gender-responsive policies to strengthen women’s empowerment, improve maternal and child health outcomes, and advance gender equality in Somalia.

## Materials and methods

### Study design and data source

This study employed a quantitative cross-sectional design using secondary data from the 2020 Somalia Demographic and Health Survey (SDHS). The SDHS provides nationally representative data on population health and demographic indicators. The design allows estimation of the prevalence of women’s healthcare decision-making autonomy and identification of associated factors.

### Study setting

The study was conducted in Somalia, a country situated in the Horn of Africa, currently navigating a complex period of post-conflict reconstruction and state-building. The nation is administratively divided into 16 regions, all of which were captured in the 2020 SDHS to ensure a comprehensive national evidence base [15]. Somalia’s demographic landscape is uniquely challenging, with a population of approximately 17 million and a significant nomadic component (38.3%), which necessitates highly adaptive and mobile health service delivery models [14, 15].

The country faces profound maternal health challenges, characterized by a high total fertility rate of 6.2 births per woman and a maternal mortality ratio of 692 deaths per 100,000 live births among the highest in the world [13]. The national health system is a fragmented mosaic of public, private, and non-governmental services, often operating in parallel due to decades of weak central governance and recurring humanitarian crises [14]. In this fragile context, access to formal healthcare remains limited, particularly in rural and nomadic areas, where traditional healers and patriarchal, clan-based social structures continue to play a primary role in health-seeking behaviors and household decision-making processes [13, 15].

### SDHS data collection

Fieldwork was conducted from January 2018 to February 2019 for the survey released in 2020 through nationally representative household surveys. Trained enumerators used standardized questionnaires covering a wide range of demographic and health indicators. The Woman’s Questionnaire, completed by women aged 15–49, served as the primary data source, including sections on reproductive history, antenatal care, and vaccination.

### Study population and sampling

**Fig. 1:**
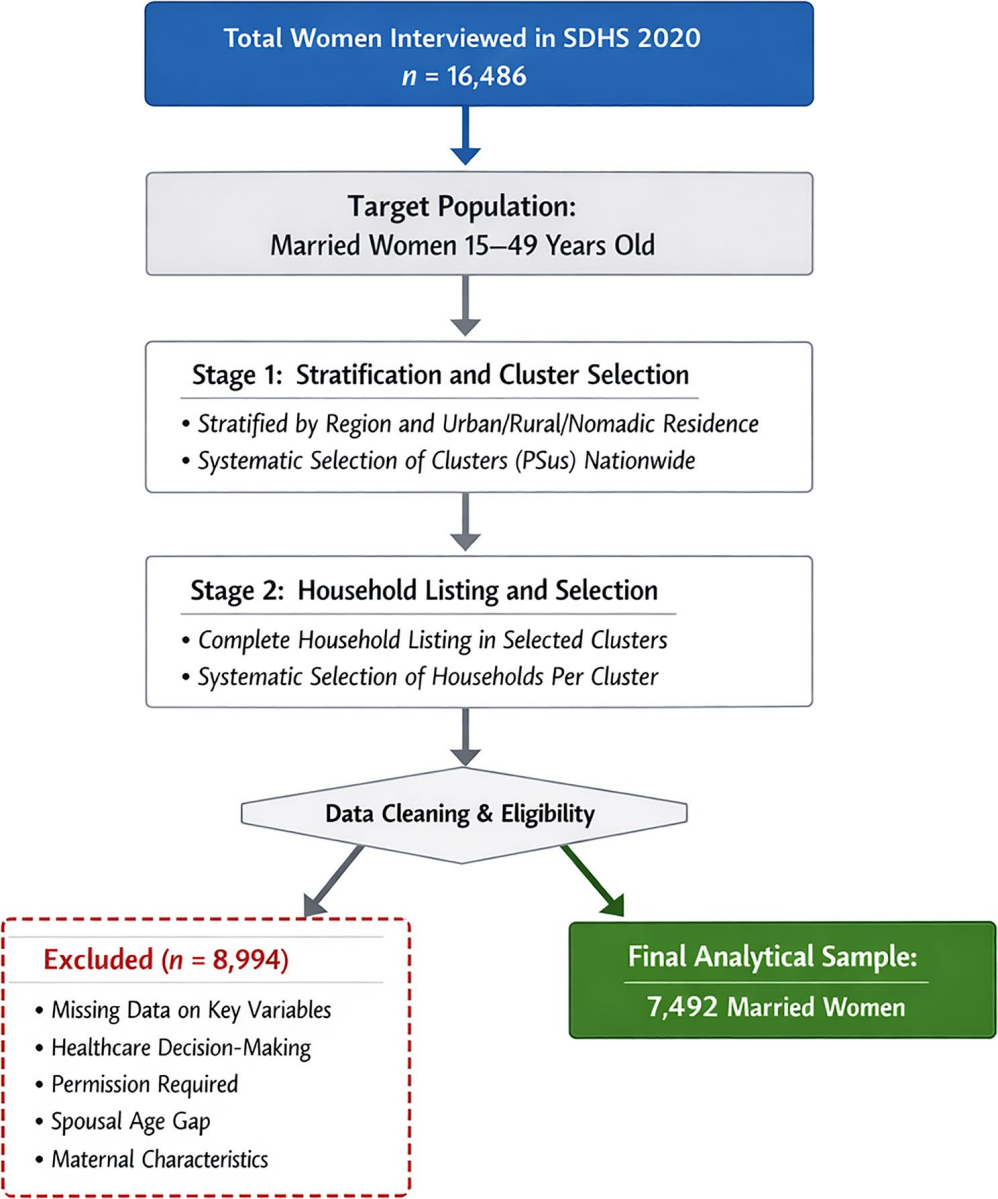
The sampling process is detailed in Fig. 1

The study population comprised married women of reproductive age (15–49 years). The 2020 SDHS utilized a multi-stage stratified cluster sampling methodology. In the first stage, clusters (Primary Sampling Units) were selected from each stratum (region and residence type). In the second stage, households were systematically selected within each cluster. After listwise deletion of cases with missing data on maternal health factors and structural barriers, the final analytical sample consisted of 7,492 married women. To ensure the findings accurately reflect the national population distribution and to compensate for the complex survey design, appropriate sampling weights were applied throughout all stages of the statistical analysis:

The sampling process is detailed in Fig. 1.

### Variables of the study

#### Outcome variable

The primary outcome is women’s healthcare decision-making autonomy. To align with a rights-based framework and the concept of relational autonomy which recognizes shared spousal decision-making as a positive expression of agency in collectivist societies. This shift adopts a relational autonomy framework, recognizing shared decisions as a positive expression of agency [3, 15]. The primary outcome was recoded into a binary variable:

1. Autonomous: Decisions made by the woman “Alone” or “Jointly with her partner.”
2. Not Autonomous: Decisions made by the “Partner alone” or “Other persons.”

### Explanatory variables

Based on a comprehensive review of existing literature and established conceptual frameworks of women’s empowerment, a multi-dimensional set of individual, household, and community-level variables were selected. Explanatory variables included individual traits, household dynamics (including the spousal age gap), and maternal health factors (ANC visits, delivery place, and birth order). Crucially, we included ‘perceived barrier: getting permission to seek care’ as a structural predictor to capture the gatekeeping dynamics prevalent in the Somali context [4, 13].

At the individual level, a woman’s age was categorized (15–24, 25–34, 35–49 years), as maturity and life experience are consistently linked to increased bargaining power within the family [12, 16–18]. Educational attainment (‘No education’, ‘Primary’, ‘Secondary+’) was included as a cornerstone of empowerment, theoretically enhancing a woman’s ability to process health information and negotiate her needs [12, 16]. Work status (‘Not working’ vs. ‘Working’) was included to capture the influence of economic independence on self-determination [19]. Finally, parity (‘No children’ vs. ‘Has children’) was included, as the transition to motherhood often fundamentally alters a woman’s status and decision-making role within the household [7].

At the household level, we included the spousal age gap (calculated as the difference between the husband’s and wife’s age) to capture the internal power dynamics and potential hierarchy within the marital union, as requested by recent rights-based frameworks [13, 16]. The household’s economic status was measured using the DHS wealth index, categorized into ‘Poor,’ ‘Middle,’ and ‘Rich’ terciles, to assess how command over economic resources correlates with individual autonomy [10, 17]. Maternal and health service utilization factors were also integrated into the model, including the number of Antenatal Care (ANC) visits (‘No visits’, ‘1–3 visits’, ‘4 + visits’), place of delivery (‘Home/Other’, ‘Public facility’, ‘Private facility’), and birth order. These variables are included not only as predictors but also as behavioral indicators of agency; prior research establishes a bi-directional link where a woman’s autonomy enables the utilization of these services, which in turn reinforces her personal agency [7, 13].

Finally, to account for the broader social context in the multilevel analysis, two key community-level variables were included: place of residence (‘Urban,’ ‘Rural,’ ‘Nomadic’) and region of residence. These variables are essential for capturing the influence of macro-level socio-cultural norms, clan-based structures, security conditions, and regional variations in health infrastructure that significantly shape the environment in which Somali women exercise their rights [11, 16].

### Statistical analysis

Statistical analyses were performed using Stata version 16.0. A multilevel mixed-effects logistic regression model was employed to account for the hierarchical structure of the SDHS data, where individuals are nested within primary sampling units (PSUs). To ensure national representativeness within this multilevel framework, a two-stage weighting procedure was implemented, setting cluster-level weights to 1 and individual-level weights to the SDHS-provided value (V005). Variables demonstrating statistical significance at *p* < 0.20 in the bivariate analysis, alongside those with established theoretical relevance, were considered for inclusion in the final multilevel model to ensure a comprehensive control of confounders. Results are reported as Adjusted Odds Ratios (AOR) with 95% Confidence Intervals (CI), and the Intraclass Correlation Coefficient (ICC) was calculated to quantify the proportion of variance in healthcare autonomy attributable to community-level factors.

The adoption of this multilevel approach is a methodological necessity in the Somali context, moving beyond individual traits to account for geospatial heterogeneity. By partitioning variance between individual agency and community-level contexts, this analysis provides an estimation of how unobserved communal norms and regional security dynamics influence a woman’s ability to exercise her health rights. Furthermore, the transition to a binary outcome—bundling sole and joint decision-making—reflects a shift toward a relational autonomy framework. In collectivist and patriarchal societies, this approach recognizes shared spousal decisions as an expression of negotiated agency, aligning with rights-based maternal health strategies that view spousal collaboration as a step toward equitable healthcare access.

### Ethical considerations

This study is based on a secondary analysis of publicly available, anonymized data from the Demographic and Health Surveys (DHS) program. The original 2020 SDHS protocol was reviewed and approved by the relevant institutional review boards in Somalia and the ICF Institutional Review Board. Informed consent was obtained from all participants before the original data collection. As the data used in this study were fully anonymized and publicly accessible, no further ethical approval was required.

## Results

The final analysis comprised a nationally representative sample of 7,492 married women. Overall, 67.1% (*n* = 5,031) of women were classified as ‘Autonomous,’ while 32.9% (*n* = 2,462) were ‘Non-autonomous.’ Table 1 presents the sociodemographic and health characteristics of the sample. In the multilevel mixed-effects model (Table 3), women with secondary education or higher had lower odds of being autonomous compared to those with no education (AOR = 0.59; 95% CI: 0.43–0.80; *p* = 0.001). Women who reported that getting permission was a big problem had higher odds of being autonomous (AOR = 1.79; 95% CI: 1.43–2.23; *p* < 0.001). Regional context emerged as a significant predictor, with women in Bakool showing the highest odds of autonomy compared to those in Awdal (AOR = 4.47; *p* = 0.001).

The final analysis comprised a nationally representative sample of 7,492 married women. Table 1 illustrates a landscape of profound socioeconomic vulnerability and structural dependency. A staggering 84.2% (*n* = 6,308) of women had no formal education, and nearly the entire sample (98.9%) was excluded from paid employment, indicating a deep-seated economic and informational marginalization.

Maternal health engagement was notably low; 65.5% of women reported no antenatal care (ANC) visits during their last pregnancy, and 76.5% delivered at home. Regarding the primary outcome, while 67.1% (*n* = 5,031) of women were classified as “Autonomous” (participating in decisions either alone or jointly), a significant third of the population (32.9%) remained entirely excluded from choices regarding their own healthcare. Crucially, 45.2% of women identified obtaining permission as a major structural barrier to accessing medical help.

**Table 1:** Sociodemographic, Household, and Health Characteristics of Respondents

Variable	Category	Unweighted *n* (%)	Weighted *n* (%)
Healthcare Decision-Making Autonomy	Non-autonomous	2,462 (32.9)	2,465 (33)
	Autonomous (Self/Joint)	5,030 (67.1)	5,027 (67)
Age Category (Years)	15–24	2,022 (27.0)	2,020 (27)
	25–34	3,599 (48.0)	3,600 (48)
	35–49	1,871 (25.0)	1,872 (25)
Education Level	No education	6,308 (84.2)	6,310 (84)
	Primary	877 (11.7)	875 (12)
	Secondary+	307 (4.1)	307 (4)
Residence	Urban	3,032 (40.5)	3,030 (40)
	Rural	2,035 (27.2)	2,037 (27)
	Nomadic	2,425 (32.4)	2,425 (32)
Wealth Index	Poor	3,439 (45.9)	3,440 (46)
	Middle	1,410 (18.8)	1,408 (19)
	Rich	2,643 (35.3)	2,644 (35)
Work Status	Not working	7,413 (98.9)	7,410 (99)
	Working	79 (1.1)	82 (1)
Has Children	No children	21 (0.3)	22 (0)
	Has children	7,471 (99.7)	7,470 (100)
ANC Visits	No Visits	4,906 (65.5)	4,905 (66)
	1–3 Visits	1,955 (26.1)	1,956 (26)
	4 + Visits	631 (8.4)	631 (8)
Place of Delivery	Home/Other	5,729 (76.5)	5,730 (77)
	Public Facility	1,408 (18.8)	1,405 (19)
	Private Facility	355 (4.7)	357 (4)
Birth Order	1st Birth	837 (11.2)	835 (11)
	2nd–3rd	2,193 (29.3)	2,195 (29)
	4th+	4,462 (59.6)	4,462 (60)
Permission Barrier to Seeking Care	Not a Big Problem	4,105 (54.8)	4,110 (55)
	Big Problem	3,387 (45.2)	3,382 (45)

Note: Unweighted frequencies (n) represent the raw analytical sample, while weighted estimates (N and %) are design-based calculations that account for the complex sampling frame of the 2020 SDHS;

The bivariate analysis (Table 2) highlights that healthcare participation is not randomly distributed but is tied to socioeconomic and behavioral factors. Women with no education were significantly less likely to be autonomous compared to their counterparts ($*p* < 0.001$). A clear “health-engagement” gradient was observed: women who attended 4 or more ANC visits and those who delivered in facilities showed significantly higher rates of autonomy ($*p* < 0.001$). Most strikingly, the “Permission Barrier” showed a powerful association; women who identified permission as a major hurdle were paradoxically more likely to be in the autonomous group, suggesting that the act of attempting to participate in decisions brings women into direct contact with traditional gatekeeping norms.

**Table 2:** Bivariate Intersections of Agency: Factors Associated with Healthcare Decision-Making Autonomy in Somalia

Variable	Category	Non-Autonomous *n* (%)	Autonomous *n* (%)	Total *n* (%)	Design-based F	*P*-value
Age (years)	15–24	701 (9)	1,282 (17)	1,983 (26)	0.35	0.703
	25–34	1,223 (16)	2,392 (32)	3,615 (48)		
	35–49	649 (9)	1,245 (17)	1,894 (25)		
Education	No education	2,089 (28)	4,265 (57)	6,354 (85)	13.47	< 0.001
	Primary	350 (5)	523 (7)	873 (12)		
	Secondary+	133 (2)	132 (2)	265 (4)		
Residence	Urban	902 (12)	1,523 (20)	2,425 (32)	1.97	0.143
	Rural	744 (10)	1,452 (19)	2,196 (29)		
	Nomadic	927 (12)	1,943 (26)	2,870 (38)		
Wealth Index	Poor	1,096 (15)	2,476 (33)	3,572 (48)	8.63	0.0002
	Middle	452 (6)	888 (12)	1,340 (18)		
	Rich	1,026 (14)	1,554 (21)	2,580 (34)		
Work Status	Not working	2,542 (34)	4,875 (65)	7,417 (99)	0.55	0.458
	Working	30 (0)	46 (1)	76 (1)		
Has Children	No children	9 (0)	18 (0)	27 (0)	0.00	0.982
	Has children	2,563 (34)	4,903 (65)	7,465 (100)		
ANC Visits	No visit	1,640 (22)	3,502 (47)	5,142 (69)	12.58	< 0.001
	1–3 visits	659 (9)	1,062 (14)	1,721 (23)		
	4 + visits	272 (4)	355 (5)	627 (8)		
Place of Delivery	Home/Other	1,891 (25)	3,902 (52)	5,793 (77)	8.28	0.0003
	Public facility	535 (7)	815 (11)	1,350 (18)		
	Private facility	144 (2)	204 (3)	348 (5)		
Birth Order	1st	272 (4)	549 (7)	821 (11)	0.14	0.858
	2nd–3rd	806 (11)	1,518 (20)	2,324 (31)		
	4th+	1,495 (20)	2,851 (38)	4,346 (58)		
Permission Barrier	Not a big problem	1,709 (23)	2,463 (33)	4,172 (56)	41.06	< 0.001
	Big problem	863 (12)	2,456 (33)	3,319 (44)		

Note: p-values derived from the Rao-Scott adjusted chi-square test. Bold indicates statistical significance at p < 0.05

The multilevel mixed-effects model (Table 3) provides the most novel insights by isolating the independent effect of each variable while accounting for community-level differences. The Permission Paradox: The strongest individual predictor was the permission barrier. Women who reported that getting permission was a big problem had 1.79 times higher odds of being autonomous (AOR = 1.79; 95% CI: 1.43–2.23; *p* < 0.001). This suggests that autonomy in Somalia is a negotiated agency; women who are actively involved in their healthcare choices are more likely to experience and report the friction of traditional gatekeeping. The Education Anomaly: Counter-intuitively, women with secondary education or higher had 41% lower odds of being autonomous compared to those with no education (AOR = 0.59; 95% CI: 0.43–0.80; *p* = 0.001). This indicates that formal schooling does not automatically translate into household bargaining power in Somalia and may even lead to a technical deference where educated women defer to male heads for complex or financial decisions. Regional Dominance: The regional context emerged as the ultimate arbiter of agency. Women in Bakool had nearly 4.5 times higher odds of autonomy (AOR = 4.47; *p* = 0.001) compared to those in Awdal, while those in Middle Shabelle (AOR = 2.37) and Hiraan (AOR = 1.97) also showed significantly higher participation. The Intraclass Correlation (ICC) confirms that community-level norms are foundational to how autonomy is exercised.

**Table 3:** Multilevel mixed-effects logistic regression of determinants of autonomy

Variable	Category	AOR	95% CI	*p*-value
Age (Ref: 15–24)	25–34	1.17	0.96–1.42	0.114
	35–49	1.12	0.85–1.48	0.427
Education (Ref: No education)	Primary	0.91	0.70–1.17	0.461
	Secondary+	0.59	0.43–0.80	0.001
Residence (Ref: Urban)	Rural	1.13	0.90–1.42	0.286
	Nomadic	0.99	0.76–1.29	0.966
Wealth Index (Ref: Poor)	Middle	0.83	0.64–1.08	0.158
	Rich	0.81	0.67–0.99	0.038
Work Status (Ref: Not working)	Working	0.76	0.41–1.39	0.365
Has Children (Ref: No)	Has children	0.96	0.35–2.64	0.939
ANC Visits (Ref: None)	1–3 visits	0.87	0.75–1.02	0.092
	4 + visits	0.83	0.62–1.11	0.201
Place of Delivery (Ref: Home/Other)	Public facility	0.95	0.79–1.15	0.616
	Private facility	0.97	0.71–1.33	0.837
Birth Order (Ref: First birth)	2nd–3rd	0.87	0.68–1.11	0.272
	4th+	0.80	0.62–1.02	0.075
Permission Barrier (Ref: Not a big problem)	Big problem	1.79	1.43–2.23	< 0.001
Age gap (continuous)	—	1.00	0.99–1.00	0.328
Region (Ref: Awdal)	Woqooyi Galbeed	1.01	0.69–1.47	0.961
	Togdheer	1.01	0.69–1.48	0.964
	Sool	0.96	0.65–1.41	0.841
	Sanaag	0.85	0.58–1.25	0.408
	Bari	1.16	0.72–1.86	0.546
	Nugaal	0.83	0.52–1.33	0.443
	Mudug	1.45	0.94–2.24	0.089
	Galgaduud	1.11	0.72–1.73	0.635
	Hiraan	1.97	1.26–3.08	0.003
	Middle Shabelle	2.37	1.52–3.71	< 0.001
	Banadir	1.86	1.21–2.86	0.005
	Bay	1.25	0.83–1.90	0.289
	Bakool	4.47	1.82–10.99	0.001
	Gedo	1.77	1.02–3.08	0.043
	Lower Juba	1.92	1.20–3.06	0.006
Random Effects	PSU-level variance (V001)	3.90e − 14	—	—
Intraclass Correlation (ICC)	PSU level	1.19e − 14	—	—

Note: AOR = Adjusted Odds Ratio; CI = Confidence Interval. The model utilizes a random intercept at the Primary Sampling Unit (PSU) level to account for unobserved community-level heterogeneity. The Intraclass Correlation Coefficient (ICC) serves as a novel metric of the neighborhood effect, quantifying the extent to which a woman’s autonomy is dictated by her regional and communal environment rather than her individual traits

## Discussion

The adoption of a binary outcome reveals that a significant third of married Somali women remain entirely excluded from healthcare decisions. By bundling sole and joint decision-making, this study recognizes shared spousal decisions as a positive expression of negotiated agency rather than a lack of independence. The ‘Permission Paradox’ identified in our findings suggests that autonomy in Somalia is a negotiated process; women who are actively involved in their healthcare choices are more likely to experience and report the friction of traditional gatekeeping. Furthermore, the inverse association between higher education and individual decision-making authority suggests a model of technical deference within certain household structures, where authority over complex matters may be transferred to male heads despite a woman’s formal schooling. The multilevel analysis confirms that autonomy is profoundly shaped by geospatial heterogeneity, where community-level norms act as a primary determinant of whether a woman can exercise her health rights.

The adoption of a binary outcome reveals that while 67.1% of married Somali women participate in healthcare decisions, a significant third (32.9%) remain entirely excluded. By bundling sole and joint decision-making, this study moves toward a relational autonomy framework, recognizing that in collectivist and patriarchal societies, shared spousal decisions are a positive expression of negotiated agency rather than a lack of independence [3, 4]. This participation rate is comparable to regional findings in Ethiopia (61.36%) but highlights that for many Somali women, agency is not an individualistic pursuit but a collaborative household process, and a meta-analysis reported rates of 61.36% in Ethiopia and 36.16% in Nigeria [17]. By conceptualizing autonomy as participation in decision-making (either sole or joint), our findings suggest that while a majority of Somali women are involved in their healthcare choices, nearly one-third remain entirely marginalized. A novel finding of this study is the ‘Permission Paradox’: women who identified obtaining permission as a major hurdle had significantly higher odds of being autonomous. This suggests that in Somalia, autonomy is a negotiated agency; women who are actively involved in their healthcare choices are more likely to experience and report the friction of traditional gatekeeping. Conversely, those entirely excluded from the process may not perceive permission as a negotiable obstacle, as decision-making power resides entirely outside their sphere of influence [4, 13]. Conversely, those who are entirely non-autonomous may not even perceive permission as a negotiable obstacle, as the decision-making power resides entirely outside their sphere of influence. This underscores that structural barriers and individual agency are not mutually exclusive but are often in direct tension during the health-seeking process [15].

The descriptive data in Table 1 provides a clear contextual backdrop for this low autonomy. With 84.81% of women having no formal education and 98.99% not engaged in paid employment, their economic and informational dependency is profound. This aligns with a vast body of literature which establishes education and economic independence as foundational pillars of female empowerment and autonomy [4, 8, 10]. Furthermore, the high prevalence of women in nomadic (38.31%) and rural (29.34%) settings, coupled with low engagement in formal health services (68.63% had no ANC visits), creates a scenario where women are physically and socially isolated, further limiting their agency.

The multilevel mixed-effects analysis confirms that autonomy is profoundly shaped by geospatial heterogeneity Table 3. The Intraclass Correlation Coefficient (ICC) indicates that a significant portion of the variance in autonomy is attributable to community-level factors rather than individual traits. The massive variation in odds across regions—with women in Bakool having nearly 4.5 times higher odds of autonomy than those in Awdal—reflects the localized influence of customary laws (Xeer), clan-based social structures, and varying levels of humanitarian health infrastructure [14, 19]. In fragile states like Somalia, the “cluster” environment (security, proximity to facilities, and communal norms) acts as a primary determinant of whether a woman can exercise her health rights.

Place of Residence also emerged as a critical predictor in the multivariate model. Nomadic women were significantly more likely than their urban counterparts to engage in joint decision-making (AOR = 1.88; 95% CI: 1.23–2.89, *p* < 0.01) and to have decisions made by others (AOR = 1.56; 95% CI: 1.04–2.36, *p* < 0.05) when compared to women making decisions alone. This suggests two distinct dynamics. The higher likelihood of joint decision-making may reflect the interdependent nature of the nomadic family unit, where survival necessitates collaboration. However, the increased influence of “Others” points to the stronger hold of traditional, patriarchal, and collectivist norms in non-urban settings, where individual autonomy is less valued than communal consensus, a pattern observed in other traditional societies [6, 15, 18].

One of the most complex findings of this study, illuminated in Table 3, is the nuanced role of education. Counter-intuitively, higher education was associated with reduced odds of autonomy. This suggests a model of ‘technical deference’ within traditional Somali households, where authority over complex or financial health matters may be transferred to male heads despite a woman’s formal schooling. This challenges the normative assumption that education uniformly enhances individual agency across all cultural contexts. This suggests a “technical deference” model where, in traditional Somali households, formal schooling may not yet translate into increased bargaining power. Instead, educated women may defer to male heads for complex or financial decisions, or they may reside in households where healthcare is viewed as a collective responsibility, leading to a different interpretation of survey questions regarding “sole” vs. “joint” control [8, 10]. This finding challenges the normative assumption that education uniformly enhances individual agency in all cultural contexts.

The inclusion of the spousal age gap and maternal health factors provides a more nuanced view of household power. While the age gap did not reach statistical significance as a main effect, the strong association between facility-based delivery, ANC utilization, and autonomy (*p* < 0.001) suggests that health-seeking behavior and agency are mutually reinforcing. Women who engage with the formal health system appear to gain the confidence or social capital required to participate in household decisions. Furthermore, the finding that working women remain more likely to be autonomous (Table 2) affirms that economic independence remains a foundational pillar of empowerment, even in settings with extreme economic marginalization Table 1, yet for this small subgroup, for this small subgroup, economic activity provides a pathway to greater self-determination, a finding consistent with literature from Senegal and Tanzania [4, 12, 18]. The nuanced finding that women in the middle wealth quintile were less likely to decide jointly (AOR = 0.66; 95% CI: 0.47–0.91, *p* < 0.05) compared to the poorest women might suggest a subtle shift in household power dynamics as wealth increases, potentially granting women more individual agency before decisions are entirely ceded to “Others.” In the final adjusted model, the woman’s own age and overall household wealth (as initially noted in the bivariate analysis) lost their direct statistical significance as main effects, suggesting their influences are mediated by these more powerful structural factors like education, residence, work status, parity, and particularly region.

Collectively, the findings of this study offer a granular, evidence-based understanding of women’s healthcare decision-making autonomy in Somalia. Far from a homogenous landscape, our results demonstrate that autonomy is profoundly shaped by an intricate interplay of individual agency, household dynamics, and, most critically, community-level and regional contexts. The persistent low prevalence of sole decision-making, coupled with the varied and sometimes paradoxical influences of factors like education and wealth, underscores the need for highly contextualized interventions.

## Conclusion

In conclusion, this study provides nationally representative evidence on the multifaceted determinants of women’s healthcare decision-making autonomy in Somalia. By adopting a relational autonomy framework, the findings demonstrate that while approximately two-thirds of married Somali women participate in healthcare decisions, one-third remain excluded from choices concerning their own health. The analysis indicates that autonomy in this fragile and conflict-affected setting operates as a negotiated agency, shaped by the interaction between individual participation and structural gatekeeping. The Permission Paradox—where women who actively engage in decision-making are more likely to report permission as a major barrier—illustrates that agency and constraint frequently coexist and may intensify during the health-seeking process.

The multilevel modeling further confirms that community-level contexts and regional norms exert a substantial influence on women’s autonomy, often exceeding the explanatory power of individual socioeconomic characteristics. The inverse association between higher education and individual decision-making authority suggests the presence of technical deference within certain household structures, where authority over complex or financial matters may be transferred to male heads despite women’s formal schooling. Collectively, these findings underscore that advancing women’s autonomy in Somalia requires context-sensitive strategies that address entrenched structural and normative barriers—particularly the expectation of familial approval—while strengthening the reinforcing relationship between formal health service utilization and participatory agency.

### Policy implications

The substantial cluster-level variance observed in the multilevel model indicates that centralized, uniform policy responses are unlikely to be effective. Interventions should be decentralized and community-driven, tailored to the distinct sociocultural and regional dynamics observed across Somali regions. Engagement with regional authorities, traditional elders, and religious leaders is essential to transform the social norm of “permission” from a restrictive gatekeeping mechanism into a supportive and collaborative household practice. In rural and nomadic settings, programs should strengthen equitable spousal partnerships within existing collectivist frameworks rather than imposing externally derived models of sole decision-making authority.

Educational and economic empowerment initiatives must also be recalibrated to address intra-household power hierarchies. The finding that formal education does not automatically translate into bargaining power highlights the need for gender-transformative curricula that incorporate rights awareness, negotiation skills, and critical engagement with household norms. Moreover, the demonstrated association between facility-based delivery, antenatal care utilization, and autonomy suggests an opportunity to embed empowerment components within maternal health services. Integrating maternal health platforms with livelihood interventions, such as microfinance and vocational training, may enhance women’s economic leverage and enable the translation of health knowledge into effective decision-making agency, particularly among marginalized rural and nomadic populations.

## Data Availability

The 2020 Somalia Demographic and Health Survey (SDHS) dataset used in this study is publicly available but requires registration. The 2020 Somalia Demographic and Health Survey (SDHS) dataset is publicly available upon registration via the DHS Program website.

## Abbreviations

ANC: Antenatal Care
AOR: Adjusted Odds Ratio
CAPI: Computer-Assisted Personal Interviewing
CI: Confidence Interval
DHS: Demographic and Health Survey
PSU: Primary Sampling Unit
RRR: Relative Risk Ratio
SDHS: Somalia Demographic and Health Survey
SE: Standard Error
UNFPA: United Nations Population Fund
